# Levothyroxine in euthyroid pregnant women with thyroid peroxidase antibody: a systematic review and meta-analysis of randomized controlled trials

**DOI:** 10.61622/rbgo/2025rbgo75

**Published:** 2025-11-18

**Authors:** Henrique Provinciatto, Maria Esther Barbalho, Pedro Matos da Câmara, Marina Simão Bertani, Alice Deberaldini Marinho, Chris Elizabeth Philip, Caroline Cristine Almeida Balieiro, Karina Felippe Monezi Pontes, Edward Araujo Júnior

**Affiliations:** 1 Centro Universitário Barão de Mauá Department of Medicine Ribeirão Preto SP Brazil Department of Medicine, Centro Universitário Barão de Mauá, Ribeirão Preto, SP, Brazil.; 2 Universidade Potiguar Department of Medicine Natal RN Brazil Department of Medicine, Universidade Potiguar, Natal, RN, Brazil.; 3 Universidade Federal do Rio Grande do Norte Department of Medicine Natal RN Brazil Department of Medicine, Universidade Federal do Rio Grande do Norte, Natal, RN, Brazil.; 4 Universidade de Caxias do Sul Department of Medicine Caxias do Sul RS Brazil Department of Medicine, Universidade de Caxias do Sul, Caxias do Sul, RS, Brazil.; 5 Universidade Federal do Estado do Rio de Janeiro Department of Medicine Rio de Janeiro RJ Brazil Department of Medicine, Universidade Federal do Estado do Rio de Janeiro, Rio de Janeiro, RJ, Brazil.; 6 Beaumont Hospital Department of Gynecology Dublin Ireland Department of Gynecology, Beaumont Hospital, Dublin, Ireland.; 7 Universidade Estadual do Amazonas Department of Medicine Manaus AM Brazil Department of Medicine, Universidade Estadual do Amazonas, Manaus, AM, Brazil.; 8 Universidade Federal de São Paulo Escola Paulista de Medicina Department of Obstetrics São Paulo SP Brazil Department of Obstetrics, Escola Paulista de Medicina, Universidade Federal de São Paulo, São Paulo, SP, Brazil.; 9 Hospital Ipiranga São Paulo SP Brazil Service of Gynecology and Obstetrics, Hospital Ipiranga, São Paulo, SP, Brazil.; 10 Universidade Municipal de São Caetano do Sul São Caetano do Sul SP Brazil Discipline of Woman Health, Universidade Municipal de São Caetano do Sul, São Caetano do Sul, SP, Brazil.

**Keywords:** Levothyroxine, Pregnancy, Euthyroid, Placebos

## Abstract

**Objective:**

Thyroid peroxidase antibody (TPOAb) is a prevalent condition amongst women of reproductive age and has been associated with adverse pregnancy outcomes. However, there is currently no proven treatment for euthyroid pregnant women with TPOAb. Therefore, we aimed to investigate the efficacy of levothyroxine treatment in this population.

**Methods:**

We searched PubMed, Embase and Cochrane Central from inception to or randomized controlled trials (RCTs) comparing levothyroxine with placebo or no treatment in euthyroid pregnant women who were positive for TPOAb. Our main outcomes were miscarriage, preterm birth, and live birth. We performed subgroup analysis based on recurrent pregnancy loss (RPL).

**Results:**

We included 8 RCTs comprising 1,645 pregnant women, of whom 814 (49.5%) were randomized to receive levothyroxine. Pregnant women treated with levothyroxine had significantly lower miscarriages (RR 0.78; 95% CI 0.63-0.98; p=0.035). No significant difference was found regarding pre-term birth (RR 0.78; 95% CI 0.55-1.13; p=0.189) and live birth (RR 1.05; 95% CI 0.99-1.12; p=0.097). Our subgroup analysis demonstrated a significant interaction (p=0.048) between patients with RPL (RR 1.21; 95% CI 1.03-1.42; p=0.023) and no RPL (RR 1.00; 95% CI 0.92-1.09; p=0.922).

**Conclusion:**

Levothyroxine reduced miscarriage in pregnant women with TPOAb and improved live birth rate when associated with RPL. Our subgroup analysis also provides evidence that levothyroxine may have a higher benefit for patients with a history of RPL.

**PROSPERO Registry:**

CRD42023410433

## Introduction

Thyroid peroxidase antibodies (TPOAb) are the most prevalent anti-thyroid autoantibodies, affecting approximately 5% to 14% of pregnant patients and around 15% of women during their reproductive years.^(1–3)^ Although its exact mechanism remains unclear, the presence of TPOAb is associated with an increased risk of miscarriage and preterm birth in the setting of normal thyroid function.^(4,5)^ Both adverse events not only impact the well-being of prospective parents but also lead to substantial costs for healthcare institutions.^(6)^

Negro et al.^(7)^ presented three hypotheses for the mechanism of TPOAb positivity: (1) patients with TPOAb autoimmunity may develop subclinical or clinical hypothyroidism during pregnancy; (2) TPOAb may represent a marker for autoimmune imbalance; and (3) thyroid autoimmunity may be an infertility marker and the maternal age could explain pregnancy loss in these patients.

While miscarriage is considered a common complication during pregnancy, occurring in 15% to 20% of all gestations, recurrent miscarriage has a prevalence of 1% and studies conflict regarding the role of TPOAb in these outcomes.^(3)^ Additionally, the relation between TPOAb positivity and preterm birth is considered a subject of investigation.^(8)^

Given the potential implications of TPOAb during pregnancy, therapeutic interventions have been studied to avoid adverse outcomes. Levothyroxine, a synthetic thyroid hormone, emerged as a potential option for TPOAb-positive euthyroid women. However, the efficacy of levothyroxine in this population remains controversial, with previous meta-analyses demonstrating no significant reduction in the risk of miscarriage or preterm birth.^(9–11)^ Furthermore, the 2017 American Thyroid Association (ATA) guidelines suggest there is insufficient evidence to support levothyroxine use in pregnant women with TPO-Ab positivity, mainly in those without a previous pregnancy loss.^(12)^

Nevertheless, recent randomized controlled trials (RCT) yielded conflicting results regarding the use of levothyroxine in pregnant women who experienced recurrent pregnancy loss (RPL).^(3,13)^ Therefore, we aimed to perform an updated systematic review and meta-analysis of RCTs incorporating subgroup analyses to assess the efficacy of levothyroxine in this population.

## Methods

This meta-analysis was performed in accordance with the Preferred Reporting Items for Systematic Review and Meta-Analyses (PRISMA) Statement and recommendations from Cochrane Collaboration Handbook for Systematic Reviews of Interventions.^(14,15)^ We prospectively registered our research protocol in the International Prospective Register of Systematic Reviews (PROSPERO) on April 2, 2023 (ID CRD42023410433).

We included in this meta-analysis studies that met all the following eligibility criteria: (1) RCTs; (2) comparing levothyroxine with placebo or no treatment; (3) in TPOAb-positive euthyroid women; (4) who were pregnant or intended to conceive and subsequently became pregnant; (5) aged eighteen years or more. We excluded studies with (1) no control group; (2) patients with thyroid disorders other than TPOAb; (3) overlapping populations, defined as studies recruiting from the same institution over an overlapping period; or (4) conference abstracts.

Two authors independently searched PubMed, Embase and Cochrane Library from inception to April 3, 2023. The following terms were used without filters, publication date, or language restrictions: (levothyroxine OR LT4) AND (women OR pregnant OR pregnancy) AND (euthyroid OR "normal TSH" OR "nonthyroidal illness syndrome") AND ("thyroid autoimmunity" OR "thyroid peroxidase antibody" OR "thyroid autoimmune disease" OR "anti-TPO" OR "TPOAb" OR "autoimmune thyroid disease" OR "thyroid antibody-positive" OR "thyroid Autoimmunity") AND (random OR randomized OR randomised).

The references from all included studies, previous systematic reviews and meta-analyses were also searched manually for any additional studies. Eventual conflicts were resolved by consensus among the authors. Two authors extracted the following data from selected RCTs: (1) country; (2) number of patients; (3) timing of levothyroxine use; (4) control; (5) TSH normal range; (6) mean gestational age; and (7) baseline TSH. Our study excluded data from patients who: (1) deviated from protocol; (2) lost to follow-up; (3) did not become pregnant; (4) or terminated pregnancy. We contacted study authors when data was unclear or missing.

The efficacy outcomes were analyzed as binary endpoints: miscarriage, defined as the spontaneous loss of an embryo or fetus before 20 weeks of gestation or with a fetal weight under 500 grams, and preterm birth, defined as the live birth of a fetus after complete expulsion or extraction from the mother, regardless of gestational age. Subgroup analyses were conducted for two categories: (1) women with recurrent pregnancy loss (RPL), defined as two or more previous miscarriages, and (2) women without RPL.

We evaluated the risk of bias using version 2 of the Cochrane Risk of Bias Assessment Tool (RoB-2) for RCTs, wherein each study was scored as high, moderate, or low risk of bias. The assessment was performed by two independent authors and disagreements were resolved through consensus after discussing reasons for discrepancy.

Moreover, we performed sensitivity analyses using leave-one-out, Baujat and L'abbé analyses. Publication bias was assessed through the generation of a funnel plot.

We computed risk ratios (RR) using the Inverse-Variance test for dichotomous outcomes and used 95% confidence intervals (CI) as a measure of effect size. We considered p-values of less than 0.05 to be statistically significant.

To assess heterogeneity, Cochran's Q test and I^2^ statistics were utilized. We classified I^2^ values of <25%, 25-75%, and >75% as representing low, moderate, and high heterogeneity, respectively. To account for potential disparities in both clinical and methodological aspects across studies, we applied the restricted maximum-likelihood estimator and random effects models for outcomes. Our meta-analysis was conducted using the meta package for RStudio version 4.2.2 (R Foundation for Statistical Computing, Vienna, Austria).

## Results

**Figure 1:**
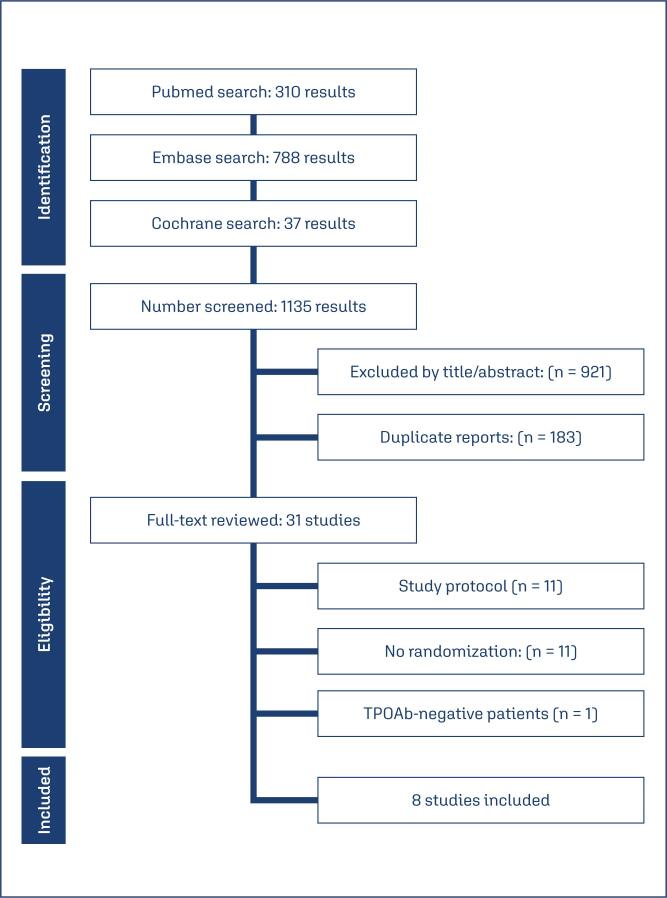
PRISMA flow diagram of study screening and selection

The initial search yielded 1,135 results. After removing duplicate records, 952 studies were screened for eligibility. Of these, 31 remained and were fully reviewed based on predefined eligibility criteria (Figure 1). Ultimately, 8 RCTs were included, comprising 1,645 patients, of whom 814 (49.5%) were assigned to the levothyroxine group. Chart 1 summarizes individual studies characteristics (3,7,8,13,16-19).

**Chart 1:** Characteristics of included trials

Study	Year	Country	Pregnant women	Starting of levothyroxine	TSH normal range (mlU/L)	TPOAb positivity (lU/mL)	Mean TSH baseline (mlU/L ± SD)
Leng et al.^(3)^	2022	China	83 vs 81	4-8 days after first prenatal visit	0.1-2.5	> 9	1.34 ± 0.71
Negro et al.^(7)^	2006	Italy	57 vs 58	3-7 days after first prenatal visit	0.27-4.2	> 100	1.6 ± 0.5
Dhillon-Smith et al.^(8)^	2019	United Kingdom	266 vs 274	Before conception	0.44-3.63	> 35-150	2.12 ± 0.91
van Dijk et al.^(13)^	2022	Netherlands	69 vs 73	Before conception	0.27-5.0	> 25-100	2.02 ± 1.01
Nazarpour et al.^(16)^	2017	Iran	18 vs 24	4-8 days after first prenatal visit	0.1-4.0	> 50	-
Negro et al.^(17)^	2016	Italy	198 vs 195	Prior to 12 weeks of pregnancy	0.5-2.5	> 16	1.37 ± 0.5
Negro et al.^(18)^	2005	Italy	24 vs 21	Before conception	0.27-4.2	> 100	1.6 ± 0.8
Wang et al.^(19)^	2017	China	99 vs 105	Before conception	0.45-4.78	> 60	2.14 ± 0.97

TSH, thyroid stimulating hormone; TPOAb, thyroid peroxidase antibody.

**Figure 2:**
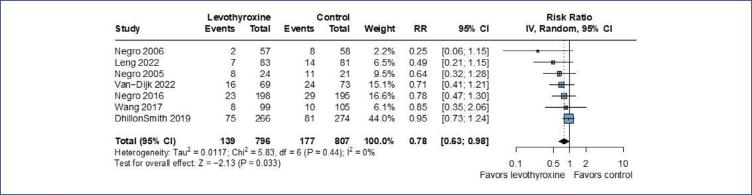
There was a significant reduction of risk of miscarriage in the levothyroxine group when compared to the control group

Seven RCTs reported miscarriage and the pooled analysis showed a significant 22% relative risk reduction in the levothyroxine group compared to the control group (RR 0.78; 95%CI 0.63 to 0.98; p=0.035; I^2^=0%; 7 RCTs; 1,603 patients (Figure 2).

**Figure 3:**
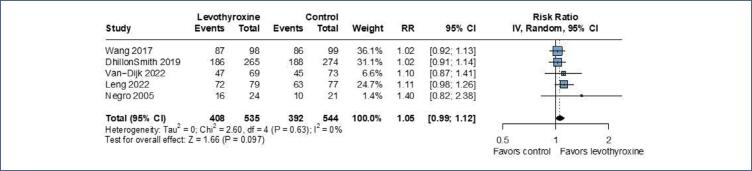
There was no significant association between live birth and levothyroxine, when compared to the control

In a pooled analysis of 5 trials, no significant effect of levothyroxine was noted for the likelihood of live birth (RR 1.05; 95% CI 0.99-1.12; p=0.097; I^2^=0%; 5 RCTs; 1,079 patients (Figure 3)

**Figure 4:**
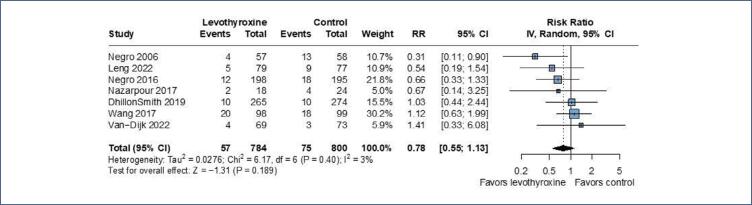
There was no significant association between preterm birth and levothyroxine, when compared to the control

Seven trials reported preterm birth and the pooled analysis showed no significant effect of levothyroxine in preterm birth (RR 0.78; 95%CI 0.55-1.13; p=0.207; I^2^=3%; 7 RCTs; 1,584 patients (Figure 4).

**Figure 5:**
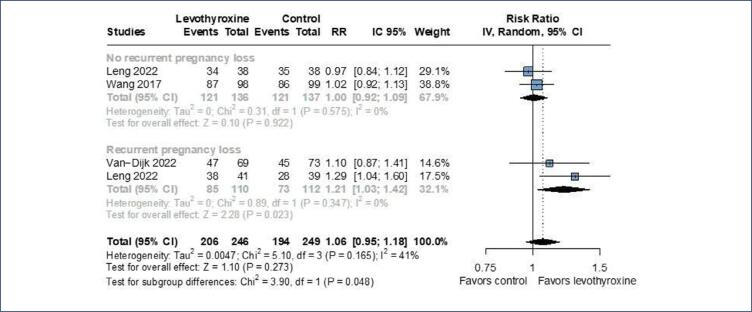
There was a significant interaction between subgroups of RPL and No RPL regarding live birth

Our subgroup analysis demonstrated a significant interaction (p=0.048) comparing the incidence of live birth between patients with RPL (RR 1.21; 95%CI 1.03-1.42; p=0.023; I^2^=0%; 2 RCTs; 242 patients and without RPL (RR 1.00; 95%CI 0.92-1.09; p=0.922); I^2^=0%; 2 RCTs; 222 patients (Figure 5).

**Figure 6:**
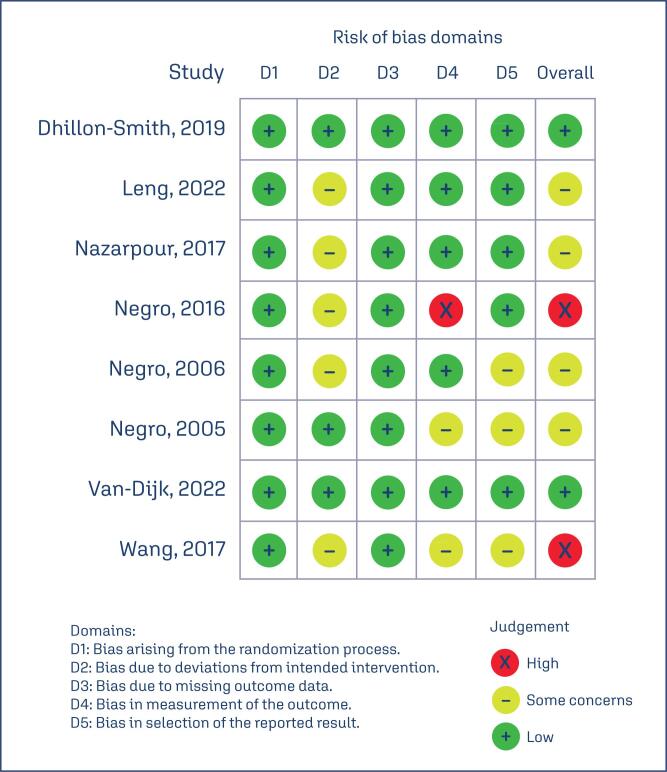
Risk of bias of included trials

Figure 6 summarizes the individual evaluation of each RCT included in the meta-analysis using the RoB 2 quality assessment tool. Two studies were rated at a high risk of bias.

We performed sensitivity analyses using leave-one-out, Baujat and L'abbé analyses for miscarriage. Publication bias was also assessed through the generation of a funnel plot.

## Discussion

In this updated systematic review and meta-analysis, we identified 1,623 patients derived from 8 RCTs comparing the use of levothyroxine with placebo or no treatment in euthyroid pregnant women with TPOAb. Our main findings were: (1) a significantly reduced risk of miscarriage; (2) no statistical difference regarding preterm birth and live birth; (3) a significant interaction of live birth based on RPL.

During pregnancy, the presence of TPOAb can yield significant implications for both the mother and the developing fetus. Untreated euthyroid women with TPOAb show a 3.9-fold risk of miscarriage and a 1.3-fold risk of preterm birth compared to TPOAb-negative subjects.^(4,5)^ Nonetheless, there is a lack of consensus regarding the efficacy of levothyroxine therapy in pregnant women with TPOAb to prevent adverse outcomes. In fact, no previous meta-analyses demonstrated the benefit of levothyroxine in this population and major guidelines reported weak or insufficient evidence to support its utilization.^(9–12)^

In contrast, one recently published RCT highlighted the advantages of levothyroxine in reducing miscarriage and improving live birth for euthyroid patients with TPOAb, particularly in those who experienced RPL.^(3)^ However, the largest study to evaluate this therapy in patients with a history of RPL revealed no such association between the intervention and both outcomes.^(13)^ Considering these conflicting results, our systematic review and meta-analysis aimed to elucidate this disparity in the existing literature and demonstrate the efficacy of levothyroxine in this population.

To the best of our knowledge, this is the first meta-analysis to demonstrate statistically significant benefits of levothyroxine in euthyroid pregnant women with TPOAb. We included recent RCTs published since prior reviews that were conducted and we have reported outcomes per clinical pregnancies, resulting in a more accurate conclusion. Our subgroup analysis also provides evidence that levothyroxine may have a higher benefit for patients with a history of RPL.

Moreover, we acknowledge the discordant results amongst studies that focused on patients with RPL. We attribute this large variation to differences in methodological approaches. Notably, Leng et al.^(3)^ used a lower cut off point for TPOAb positivity, only included patients with a TSH below 2.5 mIU/L and allocated the control group to receive no intervention rather than a placebo.

Although Leng et al.^(3)^ included patients who would not typically be classified as TPOAb-positive by employing the cut-off values used in other studies, they found a significant association between levothyroxine therapy and pregnancy outcomes in patients with RPL. This leads us to postulate the hypotheses that may be necessary to lower the appropriate cut-off for defining autoimmunity during pregnancy.

This study has limitations. The number of RCTs available for inclusion in the analysis was relatively limited, indicating a need for more well-designed and adequately powered studies to confirm the observed effects. We were not able to conduct subgroup analyses based on infertility treatment and TSH levels because of limited data. Additionally, each RCT followed the reference value for TPOAb positivity of the respective country, contributing to heterogeneity and different results.

## Conclusion

Levothyroxine effectively decreased the risk of miscarriage in unselected TPOAb-positive pregnant women. Although further research is required to establish the efficacy of levothyroxine regarding live birth and preterm birth within this population, this therapy improved live birth when associated with RPL. This suggests that levothyroxine may be a promising therapeutic intervention for TPOAb-positive patients, particularly among those who are dealing with RPL.
